# Supplementary material for: Predicting recovery outcomes following mild traumatic brain injury in an Australian community cohort: Results from the Concussion Recovery Study (CREST)
Source: PLoS One. 2026 Oct 7;21(10):e0339248. doi: 10.1371/journal.pone.0339248 (PMC13645095; doi:10.1371/journal.pone.0339248)
Supplement: Supplementary file 2 [file pone.0339248.s001.docx]

#### **S1 Table 1: Additional variables collected and examined but not included in ongoing analysis.**

| **Participant Characteristics** | **Total**  n = 232 |
| --- | --- |
| **Peri-Injury Characteristics** |  |
| **Other Injuries** |  |
| No | 126 (54.5%) |
| Yes | 105 (45.5%) |
| **Legal compensation or litigation** |  |
| No | 201 (88.5%) |
| Yes | 26 (11.5%) |
| **Post-Injury Characteristics** |  |
| **Hospital ED attended** |  |
| RPH | 94 (42.7%) |
| FSH | 17 (7.7%) |
| SCGH | 16 (7.3%) |
| Joondalup | 14 (6.4%) |
| Rockingham | 22 (10.0%) |
| SJOG Midland | 14 (6.4%) |
| SJOG Murdoch | 18 (8.2%) |
| Albany | 17 (7.7%) |
| Geraldton | 7 (3.2%) |
| Other | 1 (0.5%) |
| **CT scan conducted** |  |
| No | 100 (43.9%) |
| Yes | 128 (56.1%) |
| **Initial management** |  |
| ED | 150 (65.8%) |
| Ambulance to ED | 43 (18.9%) |
| ED and GP | 26 (11.4%) |
| GP only | 9 (3.9%) |
