# Supplementary material for: Predicting recovery outcomes following mild traumatic brain injury in an Australian community cohort: Results from the Concussion Recovery Study (CREST)
Source: PLoS One. 2026 Oct 7;21(10):e0339248. doi: 10.1371/journal.pone.0339248 (PMC13645095; doi:10.1371/journal.pone.0339248)
Supplement: Supplementary file 3 [file pone.0339248.s002.docx]

**S2 Table 2: Characteristics of eligible participants who were referred to the study but not subsequently enrolled**

| **Participant Characteristics** | **Total**  n = 136 |
| --- | --- |
| **Age (years)**, median (IQR) | 29 (22-40) |
| **Sex (female)** n(%) |  |
| Male | 83 (61.0) |
| Female | 53 (39.0) |
| **Hospital ED attended** |  |
| RPH | 56 (41.2) |
| FSH | 7 (5.1) |
| SCGH | 13 (9.6) |
| Joondalup | 4 (2.9) |
| Rockingham | 26 (19.1) |
| SJOG Midland | 16 (11.7) |
| SJOG Murdoch | 2 (1.5) |
| Albany | 9 (6.6) |
| Geraldton | 2 (1.5) |
| Other (Self-referred) | 1 (0.7) |
