# Supplementary material for: Predicting recovery outcomes following mild traumatic brain injury in an Australian community cohort: Results from the Concussion Recovery Study (CREST)
Source: PLoS One. 2026 Oct 7;21(10):e0339248. doi: 10.1371/journal.pone.0339248 (PMC13645095; doi:10.1371/journal.pone.0339248)
Supplement: Supplementary file 4 [file pone.0339248.s003.docx]

**S3 Table 3:** **Comparison of those participants who completed all follow-up timepoints (1, 3, 6 and 12 months) to those who had at least one follow-up missing**

|  | **Total** | **Completed all Follow-Ups (n = 94)** | **Follow-ups missing**  **(n = 138)** | **p-value** |
| --- | --- | --- | --- | --- |
| **Demographic Factors** |  |  |  |  |
| **Age** median (IQR) | 33 (24-50) | 36 (25-53) | 31.5 (24-48) | 0.109 |
| **Sex** n (%) |  |  |  | 0.184 |
| Female | 101 (43.5) | 36 (38.3) | 65 (47.1) |  |
| Male | 131 (56.5) | 58 (61.7) | 73 (52.9) |  |
| **Pre-Injury Factors** |  |  |  |  |
| **Previous mTBI** n (%) |  |  |  | 0.282 |
| Yes | 118 (51.1) | 44 (46.8) | 74 (54.0) |  |
| No | 113 (48.9) | 50 (53.2) | 63 (46.0) |  |
| **Previous mental health issues** n (%) |  |  |  | **0.030*** |
| Yes | 83 (35.8) | 25 (26.6) | 58 (42.0) |  |
| No | 148 (63.8) | 68 (72.3) | 80 (58.0) |  |
| **Peri-Injury Factors** |  |  |  |  |
| **Mechanism of injury** n (%) |  |  |  | 0.249 |
| Sport Related | 74 (31.9) | 34 (36.2) | 40 (28.9) |  |
| Non-sport related | 158 (68.10 | 60 (63.8) | 98 (71.1) |  |
| **Loss of Consciousness** |  |  |  | 0.939 |
| Yes | 66 (28.4) | 27 (28.7) | 39 (28.3) |  |
| No | 124 (53.5) | 51 (54.3) | 73 (52.9) |  |
| Unsure | 42 (18.1) | 16 (17.0) | 26 (18.8) |  |
| **Post-Injury Factors** |  |  |  |  |
| **PCSS Symptom Severity** median (IQR) | 29 (16-49) | 25 (14-49) | 30.5 (17-49) | 0.099 |
