# Supplementary material for: Predicting recovery outcomes following mild traumatic brain injury in an Australian community cohort: Results from the Concussion Recovery Study (CREST)
Source: PLoS One. 2026 Oct 7;21(10):e0339248. doi: 10.1371/journal.pone.0339248 (PMC13645095; doi:10.1371/journal.pone.0339248)
Supplement: Supplementary file 5 [file pone.0339248.s005.docx]

# Peer Review Report

Manuscript: Predicting recovery outcomes following mild traumatic brain injury in an Australian community cohort: results from the Concussion Recovery Study (CREST)

## General comments

This manuscript addresses an important and clinically relevant question by examining predictors of persisting post-concussion symptoms (PPCS) over 12 months in a prospective Australian community cohort. The prospective design, inclusion of community referrals beyond emergency departments, repeated follow-up, and clinically relevant outcomes (return to work, study, sport, physical activity and quality of life) are notable strengths. The statistical analyses are generally appropriate and the manuscript is clearly structured.

My may point would be to discuss the relatively high PPCS prevalence compared with some published cohorts a little more. What are the possible reasons for this? Additionally, I’m unsure why variables with less then 10 % responses were excluded from the analysis, yet they appear in the descriptive data. This should be addressed, as these factors my present useful clinical information in the predicting of PPCS and subsequent treatments.

## Specific comments

| Location | Comment |
| --- | --- |
| Page 2, line 47 | 'MF, DF, MB, DX, SR and CFP and participated...' – remove the second 'and'. |
| Page 3, line 57 | Should read ‘However,…’ |
| Page 3, line 68 | Include a space between 95%CI. This should be changed throughout the manuscript. |
| Page 5, line 116 | Use consistent terminology: either '12-month' or 'twelve-month' throughout. |
| Page 6, line 137-138 | Had all perticipants recruited be clinically diagnosed with mTBI? As the cohort includes self referrals this is not clear. |
| Page 6, line 141 | Please provide more details on how the interviews were analysed. Did you use methods such as Braun & Clarke's Reflexive Thematic Analysis to analyse the transcripts? Who checked the transcripts for accuracy? Were participants provided an opportunity to review and agree the transcripts? More detail is needed, as this is a central part of the methods used, but largely glossed over. |
| Page 6, line 144 | Brief explain the Likert scale, i.e was it o-7 or 1-7? What did 0/1 through to 7 represent e.g. no symptoms to all symptoms etc. Whilst the methods have been published previously it is useful to the reader to provide a brief summary here. |
| Page 8, line 215 | Please justify why variables with less than 10% total responses were excluded from the analysis. Whilst these responses may be small factors such as ADHD, sleep disorders etc may be useful the clinical management of PPCS. |
| Page 12, line 273 | This is a little vague. What percentage did recover by 12 months? |
| Page 13 | The statement that 96.7% returned to work despite PPCS should be interpreted cautiously because work status does not reflect work capacity. |
| Page 17, line 353 | Typographical error: 'pp=0.001' should read 'p=0.001'. |
| Page 17, line 363 | 'mTBI’s' should be written as 'mTBIs'. |
| Tables | Use consistent table formatting throughout. Suggest removing the heavy cell lines/borders. |
| Throughout | Use consistent spacing around statistical notation (e.g. 'p < 0.001' vs 'p<0.001'). |
| Throughout | Use consistent formatting of confidence intervals (95% CI) and month descriptors (3 months vs 3-months). |
| Discussion | Expand limitations relating to selection bias, attrition, and generalisability to other healthcare systems. |
