# Supplementary material for: Predicting recovery outcomes following mild traumatic brain injury in an Australian community cohort: Results from the Concussion Recovery Study (CREST)
Source: PLoS One. 2026 Oct 7;21(10):e0339248. doi: 10.1371/journal.pone.0339248 (PMC13645095; doi:10.1371/journal.pone.0339248)
Supplement: Peer review history

# Peer review: Decision Letter 0

**DOI:** 10.1371/journal.pone.0339248.r001

**Authors:** Christopher Kirk

30 Jul 2026

Dear Dr. Thorne,

Please submit your revised manuscript by Sep 13 2026 11:59PM. If you will need more time than this to complete your revisions, please reply to this message or contact the journal office at plosone@plos.org. When you're ready to submit your revision, log on to https://www.editorialmanager.com/pone/ and select the 'Submissions Needing Revision' folder to locate your manuscript file.

- A letter that responds to each point raised by the academic editor and reviewer(s). You should upload this letter as a separate file labeled 'Response to Reviewers'.
- A marked-up copy of your manuscript that highlights changes made to the original version. You should upload this as a separate file labeled 'Revised Manuscript with Track Changes'.
- An unmarked version of your revised paper without tracked changes. You should upload this as a separate file labeled 'Manuscript'.

If you would like to make changes to your financial disclosure, please include your updated statement in your cover letter. Guidelines for resubmitting your figure files are available below the reviewer comments at the end of this letter.

If applicable, we recommend that you deposit your laboratory protocols in protocols.io to enhance the reproducibility of your results. Protocols.io assigns your protocol its own identifier (DOI) so that it can be cited independently in the future. For instructions see: https://journals.plos.org/plosone/s/submission-guidelines#loc-laboratory-protocols. Additionally, PLOS ONE offers an option for publishing peer-reviewed Lab Protocol articles, which describe protocols hosted on protocols.io. Read more information on sharing protocols at https://plos.org/protocols?utm_medium=editorial-email&utm_source=authorletters&utm_campaign=protocols.

As the corresponding author, your ORCID iD is verified in the submission system and will appear in the published article. PLOS supports the use of ORCID, and we encourage all coauthors to register for an ORCID iD and use it as well. Please encourage your coauthors to verify their ORCID iD within the submission system before final acceptance, as unverified ORCID iDs will not appear in the published article. *Only*  the individual author can complete the verification step; PLOS staff *cannot*  verify ORCID iDs on behalf of authors.

We look forward to receiving your revised manuscript.

Kind regards,

Christopher Kirk, PhD, FCASES

Academic Editor

PLOS One

Journal Requirements:

When submitting your revision, we need you to address these additional requirements.

1. Please ensure that your manuscript meets PLOS ONE's style requirements, including those for file naming. The PLOS ONE style templates can be found at https://journals.plos.org/plosone/s/file?id=wjVg/PLOSOne_formatting_sample_main_body.pdf and https://journals.plos.org/plosone/s/file?id=ba62/PLOSOne_formatting_sample_title_authors_affiliations.pdf

2. Thank you for stating the following financial disclosure:

[CREST was funded through a Neurotrauma Research Program (NRP) grant. Authors MF, DF, MB, DX, SR and CFP conceptualised the study and obtained research funding.].

Please state what role the funders took in the study.  If the funders had no role, please state: "The funders had no role in study design, data collection and analysis, decision to publish, or preparation of the manuscript."

If this statement is not correct you must amend it as needed.

Please include this amended Role of Funder statement in your cover letter; we will change the online submission form on your behalf.

3. Please remove any funding-related text from the manuscript. Please note that funding information should not appear in any section or other areas of your manuscript. We will publish the funding statement that is present in the Financial Disclosure section of the online submission form.

4. Thank you for stating the following in the Competing Interests section:

[Prof. Melinda Fitzgerald declares her role as CEO of Connectivity Traumatic Brain Injury Australia Ltd. All other authors of this manuscript have no conflicts of interest associated with this publication.].

Please confirm that this does not alter your adherence to all PLOS ONE policies on sharing data and materials, by including the following statement: "This does not alter our adherence to PLOS ONE policies on sharing data and materials.” (as detailed online in our guide for authors http://journals.plos.org/plosone/s/competing-interests).  If there are restrictions on sharing of data and/or materials, please state these. Please note that we cannot proceed with consideration of your article until this information has been declared.

Please include your updated Competing Interests statement in your cover letter; we will change the online submission form on your behalf.

5. We note that you have indicated that there are restrictions to data sharing for this study. For studies involving human research participant data or other sensitive data, we encourage authors to share de-identified or anonymized data. However, when data cannot be publicly shared for ethical reasons, we allow authors to make their data sets available upon request. For information on unacceptable data access restrictions, please see http://journals.plos.org/plosone/s/data-availability#loc-unacceptable-data-access-restrictions.

Before we proceed with your manuscript, please address the following prompts:

a) If there are ethical or legal restrictions on sharing a de-identified data set, please explain them in detail (e.g., data contain potentially identifying or sensitive patient information, data are owned by a third-party organization, etc.) and who has imposed them (e.g., a Research Ethics Committee or Institutional Review Board, etc.). Please also provide contact information for a data access committee, ethics committee, or other institutional body to which data requests may be sent.

b) If there are no restrictions, please upload the minimal anonymized data set necessary to replicate your study findings to a stable, public repository and provide us with the relevant URLs, DOIs, or accession numbers. Please see http://www.bmj.com/content/340/bmj.c181.long for guidelines on how to de-identify and prepare clinical data for publication. For a list of recommended repositories, please see https://journals.plos.org/plosone/s/recommended-repositories. You also have the option of uploading the data as Supporting Information files, but we would recommend depositing data directly to a data repository if possible.

Please update your Data Availability statement in the submission form accordingly.

6. Please amend the manuscript submission data (via Edit Submission) to include author Carmela Pestell.

7. Your ethics statement should only appear in the Methods section of your manuscript. If your ethics statement is written in any section besides the Methods, please delete it from any other section.

8. We notice that your supplementary figures are uploaded with the file type “Figure”. Please amend the file type to “Supporting Information”. Please ensure that each Supporting Information file has a legend listed in the manuscript after the references list. http://journals.plos.org/plosone/s/supporting-information.

9. We notice that your supplementary tables are included in the manuscript file. Please remove them and upload them with the file type “Supporting Information”. Please ensure that each Supporting Information file has a legend listed in the manuscript after the references list.

If the reviewer comments include a recommendation to cite specific previously published works, please review and evaluate these publications to determine whether they are relevant and should be cited. There is no requirement to cite these works unless the editor has indicated otherwise.

Please review your reference list to ensure that it is complete and correct. If you have cited papers that have been retracted, please include the rationale for doing so in the manuscript text, or remove these references and replace them with relevant current references. Any changes to the reference list should be mentioned in the rebuttal letter that accompanies your revised manuscript. If you need to cite a retracted article, indicate the article’s retracted status in the References list and also include a citation and full reference for the retraction notice.

[Note: HTML markup is below. Please do not edit.]

Reviewers' comments:

Reviewer's Responses to Questions

**Comments to the Author**

1. Is the manuscript technically sound, and do the data support the conclusions?

Reviewer #1: Yes

Reviewer #2: Yes

Reviewer #3: Yes

**********

2. Has the statistical analysis been performed appropriately and rigorously?

Reviewer #1: Yes

Reviewer #2: I Don't Know

Reviewer #3: Yes

**********

3. Have the authors made all data underlying the findings in their manuscript fully available?

The PLOS Data policy

Reviewer #1: Yes

Reviewer #2: Yes

Reviewer #3: Yes

**********

4. Is the manuscript presented in an intelligible fashion and written in standard English?

Reviewer #1: Yes

Reviewer #2: Yes

Reviewer #3: Yes

**********

Reviewer #1: Dear authors, thank you very much for your valuable contribution to the journal.

This is a very interesting and timely work on a key aspect of accidentology, which remains a constant and highly relevant concern in public health worldwide. We appreciate your efforts in addressing this important issue.

Reviewer #2: This Manuscript by Jacinta Thorne et al. titled, “Predicting recovery outcomes following mild traumatic brain injury in an Australian community cohort: results from the Concussion Recovery Study (CREST)” seeks to study persistent post-concussion symptoms, quality of life impacts, and return to work/activity in individuals who received mild TBI out to 12 months post-injury. This is a well written and interesting study which represents its results and conclusions well, considers limitations and bias, and contributes to the body of knowledge surrounding mild TBI. While the manuscript is interesting, it would benefit from addressing the following concerns:

• What is the reasoning behind only comparing 3-and 12-month timepoints, and not including the 1- and 6-month timepoints, for the second and third aims? Was this a practical consideration or a statistical one? The reason should be stated.

• Lines 226-227 state that “Any statistically different factors identified from this analysis were included as covariates in subsequent multivariable modelling to account for their potential effect.” What factors were identified and included as covariates?

• What does the large “244” in the upper corner of the Table 1, second page denote?

• Lines 258-260 stat that “median symptom severity was higher for females than males at all timepoints,” but then states that “no differences were noted between females and males at six and or twelve months.” These statements appear to be at odds.

Reviewer #3: Manuscript: Predicting recovery outcomes following mild traumatic brain injury in an Australian community cohort: results from the Concussion Recovery Study (CREST)

General comments

This manuscript addresses an important and clinically relevant question by examining predictors of persisting post-concussion symptoms (PPCS) over 12 months in a prospective Australian community cohort. The prospective design, inclusion of community referrals beyond emergency departments, repeated follow-up, and clinically relevant outcomes (return to work, study, sport, physical activity and quality of life) are notable strengths. The statistical analyses are generally appropriate and the manuscript is clearly structured.

My may point would be to discuss the relatively high PPCS prevalence compared with some published cohorts a little more. What are the possible reasons for this? Additionally, I’m unsure why variables with less then 10 % responses were excluded from the analysis, yet they appear in the descriptive data. This should be addressed, as these factors my present useful clinical information in the predicting of PPCS and subsequent treatments.

Specific comments

Location Comment

Page 2, line 47 'MF, DF, MB, DX, SR and CFP and participated...' – remove the second 'and'.

Page 3, line 57 Should read ‘However,…’

Page 3, line 68 Include a space between 95%CI. This should be changed throughout the manuscript.

Page 5, line 116 Use consistent terminology: either '12-month' or 'twelve-month' throughout.

Page 6, line 137-138 Had all perticipants recruited be clinically diagnosed with mTBI? As the cohort includes self referrals this is not clear.

Page 6, line 141 Please provide more details on how the interviews were analysed. Did you use methods such as Braun & Clarke's Reflexive Thematic Analysis to analyse the transcripts? Who checked the transcripts for accuracy? Were participants provided an opportunity to review and agree the transcripts? More detail is needed, as this is a central part of the methods used, but largely glossed over.

Page 6, line 144 Brief explain the Likert scale, i.e was it o-7 or 1-7? What did 0/1 through to 7 represent e.g. no symptoms to all symptoms etc. Whilst the methods have been published previously it is useful to the reader to provide a brief summary here.

Page 8, line 215 Please justify why variables with less than 10% total responses were excluded from the analysis. Whilst these responses may be small factors such as ADHD, sleep disorders etc may be useful the clinical management of PPCS.

Page 12, line 273 This is a little vague. What percentage did recover by 12 months?

Page 13 The statement that 96.7% returned to work despite PPCS should be interpreted cautiously because work status does not reflect work capacity.

Page 17, line 353 Typographical error: 'pp=0.001' should read 'p=0.001'.

Page 17, line 363 'mTBI’s' should be written as 'mTBIs'.

Tables Use consistent table formatting throughout. Suggest removing the heavy cell lines/borders.

Throughout Use consistent spacing around statistical notation (e.g. 'p < 0.001' vs 'p<0.001').

Throughout Use consistent formatting of confidence intervals (95% CI) and month descriptors (3 months vs 3-months).

Discussion Expand limitations relating to selection bias, attrition, and generalisability to other healthcare systems.

**********

what does this mean?). If published, this will include your full peer review and any attached files.

If you choose “no”, your identity will remain anonymous but your review may still be made public.

**Do you want your identity to be public for this peer review?**  For information about this choice, including consent withdrawal, please see our Privacy Policy

Reviewer #1: No

Reviewer #2: No

Reviewer #3: No

**********

[NOTE: If reviewer comments were submitted as an attachment file, they will be attached to this email and accessible via the submission site. Please log into your account, locate the manuscript record, and check for the action link "View Attachments". If this link does not appear, there are no attachment files.]

To ensure your figures meet our technical requirements, please review our figure guidelines: https://journals.plos.org/plosone/s/figures

You may also use PLOS’s free figure tool, NAAS, to help you prepare publication quality figures: https://journals.plos.org/plosone/s/figures#loc-tools-for-figure-preparation.

NAAS will assess whether your figures meet our technical requirements by comparing each figure against our figure specifications.

Attachment Submitted filename: Peer_Review_CREST_Initial.docx

# Author comment: Author response to Decision Letter 1

**DOI:** 10.1371/journal.pone.0339248.r002

17 Aug 2026

Editor’s Comments:

1. Please ensure that your manuscript meets PLOS ONE's style requirements, including those for file naming. The PLOS ONE style templates can be found at https://journals.plos.org/plosone/s/file?id=wjVg/PLOSOne_formatting_sample_main_body.pdf and https://journals.plos.org/plosone/s/file?id=ba62/PLOSOne_formatting_sample_title_authors_affiliations.pdf

Author Response: The manuscript has now been formatted according to PLOS One’s style requirements as outlined in the templates attached.

2. Thank you for stating the following financial disclosure:

[CREST was funded through a Neurotrauma Research Program (NRP) grant. Authors MF, DF, MB, DX, SR and CFP conceptualised the study and obtained research funding.].

Please state what role the funders took in the study. If the funders had no role, please state: "The funders had no role in study design, data collection and analysis, decision to publish, or preparation of the manuscript."

If this statement is not correct you must amend it as needed.

Please include this amended Role of Funder statement in your cover letter; we will change the online submission form on your behalf.

Author Response: A statement regarding the Role of Funder has been included in the attached cover letter.

3. Please remove any funding-related text from the manuscript. Please note that funding information should not appear in any section or other areas of your manuscript. We will publish the funding statement that is present in the Financial Disclosure section of the online submission form.

Author Response: We have now removed the statements regarding funding from the manuscript at Line 47 and 787.

4. Thank you for stating the following in the Competing Interests section:

[Prof. Melinda Fitzgerald declares her role as CEO of Connectivity Traumatic Brain Injury Australia Ltd. All other authors of this manuscript have no conflicts of interest associated with this publication.].

Please confirm that this does not alter your adherence to all PLOS ONE policies on sharing data and materials, by including the following statement: "This does not alter our adherence to PLOS ONE policies on sharing data and materials.” (as detailed online in our guide for authors http://journals.plos.org/plosone/s/competing-interests). If there are restrictions on sharing of data and/or materials, please state these. Please note that we cannot proceed with consideration of your article until this information has been declared.

Please include your updated Competing Interests statement in your cover letter; we will change the online submission form on your behalf.

Author Response: We confirm that Prof Fitzgerald’s role as CEO of Connectivity Traumatic Brain Injury Australia Ltd has not impacted this manuscript, and does not alter our adherence to PLOS ONE policies on sharing data and materials. A statement to this effect has been included in the manuscript and within our cover letter. Please note, Prof. Fitzgerald has recently resigned from her role with Connectivity and is no longer the CEO of this organisation. As such, the affiliation has been removed from the author affiliations list and superscript numbers for all authors altered accordingly.

5. We note that you have indicated that there are restrictions to data sharing for this study. For studies involving human research participant data or other sensitive data, we encourage authors to share de-identified or anonymized data. However, when data cannot be publicly shared for ethical reasons, we allow authors to make their data sets available upon request. For information on unacceptable data access restrictions, please see http://journals.plos.org/plosone/s/data-availability#loc-unacceptable-data-access-restrictions.

Before we proceed with your manuscript, please address the following prompts:

a) If there are ethical or legal restrictions on sharing a de-identified data set, please explain them in detail (e.g., data contain potentially identifying or sensitive patient information, data are owned by a third-party organization, etc.) and who has imposed them (e.g., a Research Ethics Committee or Institutional Review Board, etc.). Please also provide contact information for a data access committee, ethics committee, or other institutional body to which data requests may be sent.

b) If there are no restrictions, please upload the minimal anonymized data set necessary to replicate your study findings to a stable, public repository and provide us with the relevant URLs, DOIs, or accession numbers. Please see http://www.bmj.cWEom/content/340/bmj.c181.long for guidelines on how to de-identify and prepare clinical data for publication. For a list of recommended repositories, please see https://journals.plos.org/plosone/s/recommended-repositories. You also have the option of uploading the data as Supporting Information files, but we would recommend depositing data directly to a data repository if possible.

Please update your Data Availability statement in the submission form accordingly.

Author Response: We have now uploaded the minimal anonymised data set necessary to replicate our study findings to Curtin University’s institutional data depository (https://doi.org/10.25917/1T24-NS12 ). The Data Availability statement in the manuscript has been updated to reflect this.

6. Please amend the manuscript submission data (via Edit Submission) to include author Carmela Pestell.

Author Response: We apologise for this oversight and have now amended the manuscript submission data to include author Carmela Pestell.

7. Your ethics statement should only appear in the Methods section of your manuscript. If your ethics statement is written in any section besides the Methods, please delete it from any other section.

Author Response: We have now removed the ethics statement from the Declarations section of the manuscript. It is now included only in the Methods section.

8. We notice that your supplementary figures are uploaded with the file type “Figure”. Please amend the file type to “Supporting Information”. Please ensure that each Supporting Information file has a legend listed in the manuscript after the references list. http://journals.plos.org/plosone/s/supporting-information.

Author Response: We have now modified this Figure to a Supporting Information file type named “S1_Fig1_symptoms.tif” and have ensured a legend is listed in the manuscript after the references list.

9. We notice that your supplementary tables are included in the manuscript file. Please remove them and upload them with the file type “Supporting Information”. Please ensure that each Supporting Information file has a legend listed in the manuscript after the references list.

Author Response: We have now removed the supplementary tables from the manuscript file, and have uploaded them as Supporting Information file types, with a legend listed in the manuscript after the references list.

10. If the reviewer comments include a recommendation to cite specific previously published works, please review and evaluate these publications to determine whether they are relevant and should be cited. There is no requirement to cite these works unless the editor has indicated otherwise.

Author Response: No requests have been made by reviewers to include specific previously published works.

11. Please review your reference list to ensure that it is complete and correct. If you have cited papers that have been retracted, please include the rationale for doing so in the manuscript text, or remove these references and replace them with relevant current references. Any changes to the reference list should be mentioned in the rebuttal letter that accompanies your revised manuscript. If you need to cite a retracted article, indicate the article’s retracted status in the References list and also include a citation and full reference for the retraction notice.

Author Response: Our reference list has been reviewed to ensure it is complete and correct. One additional reference has been added following this revision and has been mentioned in our accompanying cover letter.

Reviewers' comments:

Reviewer #1:

Dear authors, thank you very much for your valuable contribution to the journal.

This is a very interesting and timely work on a key aspect of accidentology, which remains a constant and highly relevant concern in public health worldwide. We appreciate your efforts in addressing this important issue.

Author Response: We thank the reviewer for taking the time to review this manuscript and for providing positive feedback on our work.

Reviewer #2:

This Manuscript by Jacinta Thorne et al. titled, “Predicting recovery outcomes following mild traumatic brain injury in an Australian community cohort: results from the Concussion Recovery Study (CREST)” seeks to study persistent post-concussion symptoms, quality of life impacts, and return to work/activity in individuals who received mild TBI out to 12 months post-injury. This is a well written and interesting study which represents its results and conclusions well, considers limitations and bias, and contributes to the body of knowledge surrounding mild TBI. While the manuscript is interesting, it would benefit from addressing the following concerns:

Author Response: We thank Reviewer #2 for their time and valued input into reviewing our manuscript. Your insight and suggestions are greatly appreciated for their help in improving this manuscript.

• What is the reasoning behind only comparing 3-and 12-month timepoints, and not including the 1- and 6-month timepoints, for the second and third aims? Was this a practical consideration or a statistical one? The reason should be stated.

Author Response: The inclusion of only the 3- and 12-month timepoints in analysis for the second and third aims was primarily a practical decision, as analysing and reporting all timepoints would have been impractical to summarise concisely for this manuscript. We chose to focus on the 3-month timepoint as consensus in the current literature uses this timepoint to define persisting post-concussion symptoms (PPCS). We also chose to include 12-month analyses as few studies, particularly in Australia, have reported longer term for people who have experienced mTBI.

This reasoning has now been included in the Statistical Analysis section of the Methods (Line 185 onwards) as follows:

“We chose to focus on the 3-month timepoint as consensus in the current literature uses this timepoint to define persisting post-concussion symptoms (PPCS)[6]. We also chose to include 12-month analyses as few studies, particularly in Australia, have reported longer term outcomes for people who have experienced mTBI.”

• Lines 226-227 state that “Any statistically different factors identified from this analysis were included as covariates in subsequent multivariable modelling to account for their potential effect.” What factors were identified and included as covariates?

Author Response: Thank you for this query. In this study, comparative analyses revealed no significant differences in demographic or injury-related characteristics between those who completed all follow-ups and those who did not, except for a lower prevalence of pre-existing mental health issues among individuals who completed all four follow-ups. As such, this factor was included as a covariate in multivariable modelling.

A statement to this effect had already been included in the Limitations sections of the manuscript (Line 748), however, we acknowledge that it has not been described adequately in the Methods or Results, and reference had not been made to Supplementary Table 3 where these results were presented.

The following statements have now been added to the Methods and Results:

(Line 239): As there was a significantly lower prevalence of pre-existing mental health issues among individuals who completed all four follow-ups, this factor was included as a covariate in multivariable modelling to account for its potential effect. No other demographic or injury-related characteristics were significantly different between those who completed all follow-ups and those who did not.

(Line 477): Previous mental health issues were also identified as a factor that may potentially contribute to attrition bias (Supplementary Table 3).

Other variables identified as promising predictors of PPCS are described in the Results section of the manuscript, as now noted in the revised Methods.

• What does the large “244” in the upper corner of the Table 1, second page denote?

Author Response: Thank you for noting this. The ‘244’ is a line number that has overlaid on the table in the PDF document version. These line numbers will not be present in the final version.

• Lines 258-260 stat that “median symptom severity was higher for females than males at all timepoints,” but then states that “no differences were noted between females and males at six and or twelve months.” These statements appear to be at odds.

Author Response: We thank the reviewer for pointing out this inconsistency. We have now rephrased this in Line 272 to read:

Median symptom severity was significantly higher for females initially (p<0.001), and at one month (p=0.01) and three months post mTBI (p=0.004) (Figure 1a). No significant differences were noted between females and males at six or twelve months.

Reviewer #3: Manuscript: Predicting recovery outcomes following mild traumatic brain injury in an Australian community cohort: results from the Concussion Recovery Study (CREST)

General comments

This manuscript addresses an important and clinically relevant question by examining predictors of persisting post-concussion symptoms (PPCS) over 12 months in a prospective Australian community cohort. The prospective design, inclusion of community referrals beyond emergency departments, repeated follow-up, and clinically relevant outcomes (return to work, study, sport, physical activity and quality of life) are notable strengths. The statistical analyses are generally appropriate and the manuscript is clearly structured.

My may point would be to discuss the relatively high PPCS prevalence compared with some published cohorts a little more. What are the possible reasons for this? Additionally, I’m unsure why variables with less then 10 % responses were excluded from the analysis, yet they appear in the descriptive data. This should be addressed, as these factors my present useful clinical information in the predicting of PPCS and subsequent treatments.

Author Response: We thank Reviewer 3 for taking the time to review this manuscript and for providing insightful comments and suggestions. Please see our response to your comments as follows.

We acknowledge the reviewer’s comment regarding the relatively high PPCS prevalence compared with some published cohorts. As outlined in the Discussion (Line 542) this is relatively consistent with previous literature examining mTBI cohorts in a non-athletic population, but is higher than the 31.3% prevalence of PPCS reported in a recent meta-analysis that included studies of all-cause mTBI. We have now elaborated further on possible reasons for this difference in the Limitations section (Line 709 onwards). This paragraph reads as follows:

“In this study, PPCS was defined a priori using a binarised cut-off score of six or more for males, and seven or more for females[34] at three months or beyond following injury. It is possible that this cut-off score was low for our population, who were generally older and less physically active than the sport-related concussion population upon which the PCSS scale and cut-off score was developed[31,34]. If we were to apply a more stringent definition, it is possible that less people would be considered to have PPCS. However, it is also possible that some people who were actually experiencing persisting symptoms would be missed. Future studies should endeavour to use standardised data collection instruments and definitions of PPCS and recovery, as outlined by a recent Delphi consensus statement[6].”

We thank the reviewer for their comment regarding exclusion of variables with less than 10% responses. Many of these variables are important and potentially useful clinical factors in predicting PPCS and have been taken into account into univariate logistic regression analysis as reported in Table 3. However, they were not included as covariates in multivariable modelling due to difficulties conducting multivariable logistic regression with small numbers/counts. This has now been outlined in further detail in the Statistical analysis section (Line 224 onwards), as follows:

“Variables with low cell counts (less than 10% of total responses) were not included in ongoing multivariable logistic regression analyses (pre-injury sleep disorders, ADHD, alcohol consumption at time of injury) because sparse cell counts can result in unstable parameter estimates, inflated standard errors, convergence difficulties, and separation, leading to unreliable odds ratio estimates. This approach is consistent with recommendations for logistic regression modelling when predictor categories are low (Heinze and Schemper, 2002).

Specific comments

Location Comment

Page 2, line 47 'MF, DF, MB, DX, SR and CFP and participated...' – remove the second 'and'.

Author Response: We thank the reviewer for picking up this error, which has now been corrected.

Page 3, line 57 Should read ‘However,…’

Author Response: We have now altered this sentence in Line 57 as suggested:

“Most people recover well following mild traumatic brain injury (mTBI). However, some experience persisting post-concussion symptoms (PPCS) for months or years”.

Page 3, line 68 Include a space between 95%CI. This should be changed throughout the manuscript.

Author Response: We acknowledge the reviewer’s request to include a space between 95% and CI, and have now altered this at Line 67 and throughout the manuscript (primarily in the Results section, Line 366 onwards).

Page 5, line 116 Use consistent terminology: either '12-month' or 'twelve-month' throughout.

Author Response: We have now modified the terminology throughout the manuscript to read ‘12-months’. In addition, we have also modified reference to other timepoints to 1-month, 3-months and 6-months throughout.

Page 6, line 137-138 Had all participants recruited be clinically diagnosed with mTBI? As the cohort includes self referrals this is not clear.

Author Response: We acknowledge the reviewer’s comment that the sentence describing self-referrals was unclear. Participants could also self-refer to the study but were required to have a clinical diagnosis of mTBI made by a medical practitioner prior to their enrolment. A sentence outlining this process has now been added to Line 138 as follows:

“Participants could also self-refer to the study but were required to have a clinical diagnosis of mTBI made by a medical practitioner prior to their enrolment”.

Page 6, line 141 Please provide more details on how the interviews were analysed. Did you use methods such as Braun & Clarke's Reflexive Thematic Analysis to analyse the transcripts? Who checked the transcripts for accuracy? Were participants provided an opportunity to review and agree the transcripts? More detail is needed, as this is a central part of the methods used, but largely glossed over.

Author Response: We thank the reviewer for this comment and questions. It appears that our use of the term “semi-structured telephone interview” was misleading, suggesting that a qualitative interview approach was used for this study. This component of the study was completion of a questionnaire collecting information on relevant aspects of the participants demographics, pre-injury status, the injury event and post-injury management, but was conducted verbally and in a conversational manner.

In order to avoid this confusion for other readers, we have now altered this sentence to read “Upon enrolment, participants completed a questionnaire via verbal telephone interview, providing details on demographics, the injury event, acute mTBI management, previous history of mTBI, and relevant medical history. Information was entered directly into a Redcap database by the researcher.” (Line 142 onwards).

Page 6, line 144 Brief explain the Likert scale, i.e was it o-7 or 1-7? What did 0/1 through to 7 represent e.g. no symptoms to all symptoms etc. Whilst the methods have been published previously it is useful to the reader to provide a brief summary here.

Author Response: We thank the reviewer for this suggestion to improve the clarity of the manuscript. We have now added additional sentences at Line 145 stating:

“Participants were asked to rate each symptom using a 7-point Likert scale where zero would indicate the participant had not experienced that symptom and six that they had experienced severe problems with that symptom on the day of assessment. Scores were then calculated to give the total number of symptoms out of a possible 22, and an overall symptom severity score out of a possible 132. Higher scores indicated greater symptom burden.”

Page 8, line 215 Please justify why variables with less than 10% total responses were excluded from the analysis. Whilst these responses may be small factors such as ADHD, sleep disorders etc may be useful the clinical management of PPCS.

Author Response: As outlined in a prior response, many of these variables are important and potentially useful clinical factors in predicting PPCS and have been included in univariate logistic regression analysis as reported in Table 3. However, they were not included as covariates in multivariable modelling due to difficulties conducting multivariable logistic regression with small numbers/counts. This has now been outlined in further detail in the Statistical analysis section (Line 224 onwards).

Page 12, line 273 This is a little vague. What percentage did recover by 12 months?

Author Response: We have now included the number and percentage of those who remained symptomatic, as well as those who had recovered. Please note, we also made a small correction to the number reported in the following sentence.

This section at Line 286 now reads as follows:

“At 12-months, most participants who reported PPCS at 3-months (n = 42, 51.2%) remained symptomatic, though 15 (18.3%) participants recovered. Conversely, while many participants without PPCS at 3-months stayed symptom-free, a small group (n = 12, 14.4%) reported PPCS when assessed at 12-months. These patterns highlight both the persistent and variable nature of PPCS after mTBI.”

Page 13 The statement that 96.7% returned to work despite PPCS should be interpreted cautiously because work status does not reflect work capacity.

Author Response: We thank the reviewer for this insightful comment. To clarify, the statement on p.13 reports that by three months 96.7% had returned to work, but does not make reference to PPCS. Line 322 provides further detail, stating that of the 96.7% that had RTW by three months post-injury, 47.8% were still experiencing PPCS. As we did not collect data regarding work capacity in this study we were unable to report this here. However, we refer the reviewer to a substantial paragraph already in the Discussion explaining the potential for loss in work capacity and productivity when returning to work post-mTBI (Line 553 onwards).

Page 17, line 353 Typographical error: 'pp=0.001' should read 'p=0.001'.

Author Response: We thank the reviewer for drawing this typograpical error to our attention. We have now corrected this to read ‘p = 0.001’.

Page 17, line 363 'mTBI’s' should be written as 'mTBIs'.

Author Response: This abbreviation has now been corrected to be written as ‘mTBIs’.

Tables Use consistent table formatting throughout. Suggest removing the heavy cell lines/borders.

Author Response: We have now altered all tables (including Supplementary tables) for consistency using the PLOS One Table formatting guidelines. We have removed the heavy cell lines and border as per the reviewer’s suggestion, however, have still included light cell borders throughout to ensure the formatting meets PLOS One style requirements.

Throughout Use consistent spacing around statistical notation (e.g. 'p < 0.001' vs 'p<0.001').

Author Response: This has now been corrected throughout the manuscript to include spaces before and after stated p-values (e.g. p = 0.001) and also before and after reported number of participants (e.g. n = 8).

Throughout Use consistent formatting of confidence intervals (95% CI) and month descriptors (3 months vs 3-months).

Author Response: We acknowledge the reviewer’s request to use consistent formatting of confidence intervals and month descriptors. As previously described above, these have now been altered throughout the manuscript.

Discussion Expand limitations relating to selection bias, attrition, and generalisability to other healthcare systems.

Author Response: We acknowledge the reviewer’s suggestion to expand the limitations section relating to selection bias, attrition and generalisability to other healthcare systems. As such, additions have been made to each of these sections from Line 732 onwards:

Selection Bias (Line 730):

“As the population characteristics of those who were not referred are unknown, it is not possible to determine whether our sample is a true reflection of all mTBI presentations. The demographic characteristics (age and sex) of participants who were referred to the study but not enrolled were comparable to those who were enrolled, suggesting that these cohorts were comparable. However, these comparisons were limited to the demographic information available and cannot exclude differences in symptom severity, comorbidity, socioeconomic circumstances, healthcare-seeking behaviour, or willingness and capacity to participate. In addition, the predominance of Emergency Department recruitment may have underrepresented people managed solely in primary care, those who did not seek medical assessment, and individuals facing barriers to referral or research participation. The observed prevalence and predictors of PPCS should therefore be interpreted as applying primarily to medically assessed adults who were accessible and willing to enrol, rather than to all people with mTBI in the community.”

Attrition Bias (Line 747):

“Pre-existing mental health issues were included as a covariate in multivariable modelling to account for the potential influence of this factor, though residual attrition bias may remain despite covariate adjustment. It is also not possible to fully address bias arising from other unmeasured factors related to continued participation[45]. Because follow-up completion may have differed according to symptom trajectory, functional recovery, competing demands, or capacity to engage by telephone, our results may potentially either overestimate or underestimate the frequency and determinants of PPCS.”

Generalisability (Line 755):

This study was conducted across metropolitan and regional Western Australia, with most participants recruited through Emergency Departments. Differences between healthcare systems in access to acute assessment, primary care and rehabilitation, referral pathways, and compensation arrangements may influence who presents for care, which treatments are received, and how recovery and functional outcomes are reported. Accordingly, the prevalence estimates and predictive performance of the models should be externally validated before being generalised to other Australian jurisdictions or countries, particularly in settings with different patterns of mTBI presentation and follow-up care.

Attachment Submitted filename: Response to Reviewers.pdf

# Peer review: Decision Letter 1

**DOI:** 10.1371/journal.pone.0339248.r003

**Authors:** Christopher Kirk

21 Sep 2026

<p>Predicting recovery outcomes following mild traumatic brain injury in an Australian community cohort: results from the Concussion Recovery Study (CREST)

PONE-D-25-61889R1

Dear Dr. Thorne,

We’re pleased to inform you that your manuscript has been judged scientifically suitable for publication and will be formally accepted for publication once it meets all outstanding technical requirements.

Congratulations!

Within one week, you’ll receive an e-mail detailing the required amendments. When these have been addressed, you’ll receive a formal acceptance letter and your manuscript will be scheduled for publication.

An invoice will be generated when your article is formally accepted. Please note, if your institution has a publishing partnership with PLOS and your article meets the relevant criteria, all or part of your publication costs will be covered. Please make sure your user information is up-to-date by logging into Editorial Manager at Editorial Manager® and clicking the ‘Update My Information' link at the top of the page. For questions related to billing, please contact billing support.

If your institution or institutions have a press office, please notify them about your upcoming paper to help maximize its impact. If they’ll be preparing press materials, please inform our press team as soon as possible -- no later than 48 hours after receiving the formal acceptance. Your manuscript will remain under strict press embargo until 2 pm Eastern Time on the date of publication. For more information, please contact onepress@plos.org.

Kind regards,

Christopher Kirk, PhD, FCASES

Academic Editor

PLOS One

Additional Editor Comments (optional):

Reviewers' comments:

Reviewer's Responses to Questions

**Comments to the Author**

Reviewer #2: All comments have been addressed

Reviewer #3: All comments have been addressed

**********

2. Is the manuscript technically sound, and do the data support the conclusions?

Reviewer #2: Yes

Reviewer #3: Yes

**********

3. Has the statistical analysis been performed appropriately and rigorously?

Reviewer #2: Yes

Reviewer #3: Yes

**********

4. Have the authors made all data underlying the findings in their manuscript fully available?

The PLOS Data policy

Reviewer #2: Yes

Reviewer #3: Yes

**********

5. Is the manuscript presented in an intelligible fashion and written in standard English?

Reviewer #2: Yes

Reviewer #3: Yes

**********

Reviewer #2: This manuscript titled “Predicting recovery outcomes following mild traumatic brain injury in an Australian community cohort: results from the Concussion Recovery Study (CREST)” by Jacinta Thorne et al. seeks to seeks to study persistent post-concussion symptoms, quality of life impacts, and return to work/activity in individuals who received mild TBI out to 12 months post-injury. I maintain the view that this is a well written and interesting study which substantively contributes to the body of knowledge surrounding mild TBI. The authors have been thorough in their responsiveness to previous questions and concerns and clear in their changes. The manuscript, as it exits in its revised state, is substantively improved and leaves me with no further concerns.

Reviewer #3: (No Response)

**********

what does this mean?). If published, this will include your full peer review and any attached files.

If you choose “no”, your identity will remain anonymous but your review may still be made public.

**Do you want your identity to be public for this peer review?**  For information about this choice, including consent withdrawal, please see our Privacy Policy

Reviewer #2: **Yes:**  Justin A. Courson

Reviewer #3: **Yes:**  Dr Howard Thomas Hurst

**********

# Editor report: Acceptance letter

**DOI:** 10.1371/journal.pone.0339248.r004

**Authors:** Christopher Kirk

PONE-D-25-61889R1

PLOS One

Dear Dr. Thorne,

I'm pleased to inform you that your manuscript has been deemed suitable for publication in PLOS One. Congratulations! Your manuscript is now being handed over to our production team.

At this stage, our production department will prepare your paper for publication. This includes ensuring the following:

* All references, tables, and figures are properly cited

* All relevant supporting information is included in the manuscript submission,

* There are no issues that prevent the paper from being properly typeset

You will receive further instructions from the production team, including instructions on how to review your proof when it is ready. Please keep in mind that we are working through a large volume of accepted articles, so please give us a few days to review your paper and let you know the next and final steps.

Lastly, if your institution or institutions have a press office, please let them know about your upcoming paper now to help maximize its impact. If they'll be preparing press materials, please inform our press team within the next 48 hours. Your manuscript will remain under strict press embargo until 2 pm Eastern Time on the date of publication. For more information, please contact onepress@plos.org.

You will receive an invoice from PLOS for your publication fee after your manuscript has reached the completed accept phase. If you receive an email requesting payment before acceptance or for any other service, this may be a phishing scheme. Learn how to identify phishing emails and protect your accounts at https://explore.plos.org/phishing.

If we can help with anything else, please email us at customercare@plos.org.

Thank you for submitting your work to PLOS One and supporting open access.

Kind regards,

PLOS One Editorial Office Staff

on behalf of

Dr. Christopher Kirk

Academic Editor

PLOS One
